# A qualitative study about explanatory models of alcohol use disorder among patients and relatives in a Ugandan mental hospital

**DOI:** 10.1186/s12888-024-05677-4

**Published:** 2024-03-21

**Authors:** Hanna Rudenstrand, Sofie Bäärnhielm

**Affiliations:** 1 Aleris Psychiatry Rinkeby, Stockholm, Sweden; 2 Centre for Psychiatry Research, Department of Clinical Neuroscience, Karolinska Institutet & Stockholm Health Care Services, Transcultural Centre, Region Stockholm, Stockholm, Sweden

**Keywords:** Explanatory models, Addictive behaviour, Alcohol addiction, Alcohol dependence, Alcohol use disorder, Mental health, Relatives, Culture, Qualitative research

## Abstract

**Background:**

Alcohol use disorder (AUD) is a major clinical problem in Uganda. Explanatory models (EMs) of illness are important as they have consequences for treatment. Clinicians´ knowledge about patients´ EMs can improve understanding of the latter´s perspectives and adapting treatments. There is a lack of African studies about EMs of AUD. The aim of this study was to explore EMs for AUD among hospitalized patients and their relatives at the alcohol and drug unit (ADU) at Butabika hospital in Uganda.

**Methods:**

An adapted version of the Explanatory Model Interview Catalogue (EMIC) was used for interviews with ten patients and five relatives to investigate how both hospitalized patients with AUD and their relatives understand the disease. Data were analysed for themes with a qualitative content analysis and support of the software program, OpenCode 4.03.

**Results:**

Five major themes were identified from the patient interviews: “Context promotes AUD”; “Alcohol is part of culture”; “Spiritual causes of AUD in the community”; “Help through Western medicine and religious sources is preferred” and “Social problems and stigmatization”. Six major themes identified from the interviews with relatives were: “Numerous causes of drinking alcohol”; “Devastating consequences of drinking alcohol”; “Exploiting persons with AUD”; “Others’ suffering”; “Relatives struggling for help” and “Suggested solutions”.

**Conclusions:**

Patients’ EMs of AUD included social and spiritual explanations. Alcohol is seen as an important part of the Ugandan culture among both patients and their relatives. The results indicate it is important in clinical contexts to investigate the EMs of the patients and relatives to individually tailor treatment interventions.

## Background

In Uganda the use of alcohol and other substances during adolescence and early adulthood has become a serious public health problem [1]. Alcohol consumption in Uganda is among the highest in the world at 26.0 L per capita per year in 2016 [2] and it is reported that 9.8% of the adult population has an alcohol use related disorder [3]. In Uganda, alcohol dependence is among the main causes of psychiatric morbidity [4]. Excessive consumption of alcohol has been identified as one of the major causes of poverty in Uganda, as it leads to declined productivity, increased expenditure and loss of assets, impaired judgment and vulnerability to disease thereby being a driver and maintainer of chronic poverty [5]. In Uganda, religion is an important component of life [6]. Almost all (99%) adults belong to an organized religion, with 85% identifying as Christian and 12% as Muslim. Therefore, the influence of religion, spirituality, and religiousness on alcohol use is important to explore when designing interventions to reduce levels of drinking. It is important to pay attention to individual stories, especially regarding stigmatized conditions. It is essential to understand illness from both the biomedical and lay point of view because illness explanations are not only about details of the individual’s illness experience, they also include the social context [7]. EMs are broad notions that include all the above. Differences in culture and EMs of illness between patients and clinicians may affect psychiatric care including evaluation of patterns of symptoms, help-seeking behaviour, treatment compliance, and perceived quality of care [8, 9]. Globally, there are few studies about EMs and addiction. Little attention has been paid to cultural differences in EMs of addictive behaviour [10]. In a study of those attending traditional healers in Eastern Uganda, diagnosable current mental illness was found in 60.2%, and 16.3% had had one disorder in their lifetime [11]. If the symptoms were moderate to severe, the patients were more likely to use both biomedical services and traditional healers. This makes cooperation between mental health professionals and traditional healers important. Alcohol-related problems are common among primary health care (PHC) patients in Uganda [12]. Very few of these problems are detected and treated. There is a lack of trained PHC professionals in diagnosing and treating alcohol-related problems. The primary care system in Uganda is poorly resourced, inadequately funded, and ill-equipped to address mental health concerns [13]. Nationwide, there are only 53 psychiatrists, approximately one psychiatrist per 1 million population. These psychiatrists are mostly located in urban centres and are employed as university lecturers and researchers, leaving few to serve as clinicians [14]. An estimated 200,000 traditional medicine practitioners exist in Uganda, and 60%–79% of the Ugandan population uses traditional medicine to meet their healthcare needs [15]. Traditional healers in Uganda employ a variety of methods for treating mental illness including herbs, appealing to spirits and divine intervention from gods [16, 17]. Despite alcohol drinking related problems being a major health problem in Uganda, there is limited use of pharmacological therapies in its treatment. Anecdotal evidence shows that herbal medicines are used by traditional medicine practitioners to treat alcohol drinking problems in Uganda [18].

How EMs for addictive behaviour among youths can vary culturally was found in a German study [19]. Central medical concepts, such as "physical dependence" or "reduced control of substance intake”, were found to be inadequate to characterize problems of addictive behaviour according to a notable part of Turkish youths compared to German youths in Germany. The Turkish youths had a different meaning regarding addiction compared to health professionals. The results from the study show that preventive information programs must be adapted to these differences. Concepts that are accepted and clearly associated with addictive behaviour by immigrant populations must be used [19]. In a Paris suburb, immigrants, mostly from North Africa, attributed the causes of their addiction more frequently to social and magico-religious factors and less to psychological factors than non-migrants [20]. Qualitative studies on the expectations and meanings of the use of alcohol would help us understand better the reasons why people drink and why they drink in a particular way. There are few studies on EM in and AUD from LMICs (low- and middle-income countries). However, a study from India about EMs and coping strategies for AUD found strategies like religious and spiritual strategies need to be considered by clinicians when delivering an intervention to make it contextually appropriate and acceptable [21]. In a study from Sri Lanka, reduction of stress was seen as a prevalent motive for alcohol consumption among young men [22]. These studies indicate that treatments need adjustments regarding population, language and understanding.

There is a lack of studies exploring patients and their relatives´ EMs of AUD in SSA (Sub-Saharan Africa). This leads to a risk of misunderstandings in clinical situations with patients and relatives seeking help. Learning about EMs is important to facilitate treatment and contribute to compliance. The aims of this study were to explore EMs for AUD among hospitalized patients and their relatives in Uganda.

## Methods

### Design

To explore EMs of AUD a qualitative methodology was used. An adapted version of EMIC [8] was utilized to investigate how patients with AUD and their relatives understand AUD and how they relate to the problem. EMIC is a semi-structured interview protocol that refers to a collection of locally adapted EM interviews rooted in a common framework. It covers various areas of interest: patterns of distress, perceived causes of the problem, preferences for help seeking and treatment, disabilities, and stigma. Questions about mental problems and AUD were added to adapt the form. There is one version of EMIC that is written for use with patients and another version for use with non-affected persons, in this study, relatives. Two vignettes on AUD (a man and a woman), based on clinical cases, were given to the relatives before the interview. The vignettes were created according to the DSM-5 criteria for AUD. Questions related to the cases followed according to the EMIC form and follow-up questions were asked when needed.

Participant identification codes were used to anonymize the data. Ethical approval was obtained from the School of Medicine Institutional Review Board at the College of Health Sciences Makerere University as well as the Uganda National Council for Science and Technology. Permission was received to conduct the study at the Ugandan National Mental Referral Hospital (Butabika Hospital). Informed Consent was obtained from all study participants.

### Setting

Data collection was conducted at the Ugandan National Mental Referral Hospital, located about 10 km east of the capital Kampala in Uganda. Butabika hospital is a large mental hospital in the country and the only one with specialized addiction care. There were 24 beds in the ADU at the time for the study and relatives visited frequently.

### Participants

A total of 10 patients (all men) and 5 relatives (3 men and 2 women) were recruited from the ADU at the Ugandan National Mental Referral Hospital (see Table 1). Female patients were rare on this ward and at the time for the study there were only men in the ADU. The participants were selected through purposeful sampling [23] to deliver informed findings to illuminate EMs about AUD in Uganda. All participants understood, and could communicate in English.

**Table 1:** Demographic information of participants

	**Patients**	**Relatives**
**Number of participants**	10	5
**Age (years)**		
Mean (range)	31 (22–46)	45 (28–62)
**Gender**		
Male	10	3
Female	0	2
**Marital status**		
Married	1	4
Single	7	1
Divorced	1	0
Widow	1	0
**Children**		
Yes	6	5
No	4	0
**Educational status**		
Secondary	3	1
Diploma	1	0
Bachelor or higher	5	4
Master	1	0
**Religion**		
Protestant	5	3
Catholic	3	1
Muslim	2	1

### Data collection

Data were collected by semi-structured, face-to-face individual interviews from September to December 2015 by the first author (HR). To include patients with AUD and their relatives, the first author (HR) had meetings with nurses at the ADU, Butabika hospital, to recruit informants. An informed consent form in English was administered to all the participants before the interview. It contained information about the background, purpose, potential risks, confidentiality and expected benefits of this study. A written consent was compulsory to participate. In connection with the application for an ethical permit, it was recommended by a research collaborator to compensate the participants in this study. All participants received 10 000 Ugandan Shillings (2,75 USD) for their participation in the study. The interviews took place in the ADU at Butabika hospital, and all relatives were related to patients from the ADU. The interviews were conducted in English and demographic information was registered. All information was written down verbatim in a notebook during the interviews and then typed on the computer. Each interview took 50–120 min and was finished when the EMIC form was completed. There were no dropouts during the study and none of the participants declined to participate after being given information. Data collection continued until no new information emerged.

### Data analysis

Data were analysed for themes with a qualitative content analysis [24]. Data were coded and categorized with the support of the software program OpenCode 4.03 (http://www.phmed.umu.se/enheter/epidemiologi/forskning/open-code/). Data were coded line by line as it was written in the interviews and the participants’ wording was used. The text was systematically condensed through repeated reviews and continuous coding. Memos were written to facilitate the process. Each question from all patients and relatives was analysed separately. The coding, syntheses and the following thematic content analysis were discussed with the last author (SB) during regular meetings. Similar codes were accumulated into categories. Some codes were put in several categories. Categories were merged into subthemes and themes.

## Results

Qualitative analysis of interview transcripts highlighted five themes for the patients and six themes for the relatives. These are shown in Table 2.

**Table 2:** Table of themes

Patients´ themes	Relative´s themes
Context promotes AUD	Numerous causes of drinking alcohol
Alcohol is part of culture	Devastating consequences of drinking alcohol
Spiritual causes of AUD in the community	Exploiting persons with AUD
Help through Western medicine and religious sources is preferred	Others’ suffering
Social problems and stigmatization	Relatives struggling for help
	Suggested solutions

### Context promotes AUD

Patients talked about drinking to reduce stress. They experienced an environment with peer pressure, unemployment, poor working conditions, accessibility, and cheap local alcohol that promotes alcohol consumption. Patients wanted the government to have a stricter policy concerning alcohol, with legislation regulating its accessibility and affordability.

“The government has to be stricter. Alcohol needs to be restricted. Lawmakers should intervene. Alcohol is destroying the young generation.” /A 28-year-old man.

### Alcohol is part of culture

Patients said that men are supposed to drink alcohol. Many Ugandans start drinking alcohol at a young age. Patients said that some parents give small amounts of alcohol to their children early in life. In the villages it is common to drink fermented sorghum and millet with long straws in a shared pot (“Malwa”).

“It is part of the culture to drink “Malwa”. It is nutritious. Some people take it as food. It is alcohol with a small percent.” / A 28-year-old man.

### Spiritual causes of AUD in the community

Some patients reported that others thought they were bewitched, but none of the patients believed that there could be a spiritual cause of their drinking. Some patients earlier thought that a spirit was forcing them to drink as they said that most Ugandans have such beliefs. The participating patients talked about their own experiences of seeking care from witch doctors and explained that there is a common belief in Uganda that a spirit is drinking through the person.

“I met two witch doctors. It was my mother´s idea. I thought that maybe it was some spiritual power working on me. We went there to ask if the ancestors wanted something from me. It is like forces or energy working on you or following you. The dead man's spirit is drinking alcohol through you.” /A 28-year-old man.

### Help through Western medicine and religious sources is preferred

The patients in this study sought help in churches, from relatives and friends, traditional healers or private rehabilitation centres before coming to Butabika hospital. They had also tried self-control, for example, avoiding friends and environments with alcohol. Instead, they had tried to be with people who did not drink alcohol, but motivated and encouraged them to stay sober. Others tried to practice sports or take part in church activities or domestic work, for example, cooking. Changing diet, switching to coffee, or starting to smoke a lot of cigarettes were other ways mentioned as substitutes for drinking alcohol.

A few patients had been to private rehabilitation centres before coming to Butabika hospital, but it was expensive, and it did not work for them. Most patients interviewed had tried traditional medicine before coming to the ADU. They said that in the villages, traditional healers were available and Western medicine was hard to access. The patients were now generally opposed to traditional medicine since it had not helped them, but they were positive to assistance from religious sources. Some patients had been recommended by their priest to go to rehab (ADU) for detoxification.

The patients who came to the ADU at the Butabika hospital had heard positive reports about the treatment. A contributory reason to visit ADU was also that the patients knew that an examination of the body would be carried out. Some patients feared liver disease and HIV (human immunodeficiency virus). All patients believed that medicine from Butabika hospital was beneficial. Therapy and counselling were considered more helpful than medicine, but a combination was preferred. The patients in this study used Western medicine and all were positive to it. At the ADU at Butabika hospital they had spiritual therapy every week. Representatives from different religions or, for example, AA (Alcoholics anonymous) were invited to give lectures from their organization’s, or a religious, point of view as a part of the treatment. They had different themes and could read from their religious texts. It was seen as helpful by the patients in general and participation was voluntary. Some patients visited AA meetings frequently and gave positive evaluations.

“The soothsayer told lies to me.…I got medicine and drank it with water. Nothing happened. Later I realized that it is only the hospital and the church that are sources for help.” / A 46-year-old man.

### Social problems and stigmatization

Financial problems, in many cases due to loss of job, and deteriorated relations with family and friends, were the most frequently reported effects on life for the patients. The majority reported that their alcohol problems might cause social problems for their children in the community. The behaviour of drinkers had brought shame to the patients’ families and spoiled the family´s reputation. The patients felt disrespected and rejected, even by family and friends. They had difficulties getting along with family, relatives, and friends. Some family members and friends had avoided them. It was common that other people commented to the patients about their alcohol problems. Some people were said to think that Butabika hospital only dealt with ‘madness’.

“Earnings were affected. Family members were affected. My children suffered a lot and they had to do all the household work and even cooking. The three sons are 7, 9 and 11 years old.” /A 37-year-old man.

#### Interviews with the relatives

### Numerous causes of drinking alcohol

Relatives said that if a person cannot afford alcohol, others buy it for them as it is so cheap. Alcohol is available with bars open 24/7, and some people live in bars. Advertisements about alcohol at the bars and influence from actors and artists drinking in the media also encourage people to drink alcohol. Social causes, like peer groups, loneliness, unemployment, and irresponsible parents were also mentioned as well as using alcohol to gain confidence and ‘make them someone’. Biological and psychological reasons like grief and mental disorders were also given. According to one of the relatives, both upbringing and hereditary factors could cause people to drink alcohol, for example family background of drinking alcohol or depression. Another relative said that most mental disorders came from witchcraft. Spiritual causes were reported as a cause of drinking alcohol, for example that a relative had a grudge against the person with AUD. The person could feel something is telling them to drink.

“Most patients here at the ADU at Butabika are school or university dropouts they told me here. They get disappointment, then stress, then depression, then become totally disorganized and hide in smoking and drinking. Then they cannot sleep without alcohol.” /48-year-old woman.

“We have beliefs in Uganda that somebody can do something to you, and it will affect you for the rest of your life. Because of this witchcraft they will drink for the rest of their lives.” /28-year-old man.

### Devastating consequences of drinking alcohol

Alcohol causes several problems according to the relatives. Violence, prostitution, risk of accidents, high criminal rate, physical problems like liver diseases, lung diseases and mental problems were mentioned. AUD causes social problems like poverty, unemployment, and failure to study or work. Family problems are common, with family rejection and children being left alone. Stigma was affirmed because of drinking alcohol. Others just ignore and overlook the persons with AUD and most of them are left there according to the relatives. Friends disappear completely and most people avoid the person because of the problem. The neighbourhood hates it if they see someone drunk and have no trust. Only those with caring parents get help.

“Sometimes the family doesn´t want to see them again. Some parents send the children away if they get into drugs, for example, to Butabika, leave them and walk away.” / A 53-year-old woman.

### Exploiting persons with AUD

The relatives had opinions about the government and thought that it was quiet and only wanted votes. The government open bars, and these belong to the government. Laws are not followed, and no one is arrested. The police put the drunken alcoholics in jail and someone else must come and pay a bribe to get them out. A relative reported that students can stay in private schools, even if they take drugs, since the owner wants a school fee.

“People know alcohol makes money, so they go home and produce it. The cheap alcohol is very dangerous, and it is bought by youngsters and those who don't have a lot of money.” /A 48-year-old woman.

### Others’ suffering

Others﻿ in the community think less of the family of those with AUD and this can make it harder for other family members to find marriage partners. The relatives talked about children and youngsters affected by the heavy drinking. The biggest problem in the family is if the woman drinks, as women take care of children, and it is a risk factor to start drinking alcohol if the mother sells it. The family sleep in the same room as where they sell alcohol and cigarettes. A child can go to a shop and buy alcohol.

”People leave their children suffering. Parents work all day. The children are left at home alone and it can cause addiction. Even children who are like 2, 4, 6 years old.” /A 48-year-old woman.

### Relatives struggling for help

Hospitals like Butabika hospital, rehab centres, non-governmental organizations (NGOs), traditional healers, witch doctors, religion, the community, and the family were seen as possible sources of help for AUD according to the relatives. There were generally positive opinions about medicines and counselling/therapy from Butabika hospital. The relatives thought that these patients needed to be talked to and most of the respondents thought the persons with AUD should seek help at Butabika. Most relatives addressed the problem that there is a lack of knowledge among Ugandans that Butabika hospital exists and that there is an ADU. The respondents reported that the government needs to help take persons with AUD to hospitals like Butabika. Without money there is no other health care than Butabika hospital according to relatives in this study. Without treatment the persons with AUD become mad. It is hard to give up alcohol entirely and there is no help to stop drinking in the villages. Traditional healers were not seen as good, except one relative who had seen positive effects. Treatments received from witch doctors had not been helpful.

“These days people have started making rehab centres for those who have money. Without money there is nothing… If they don´t want to quit they go out drinking again.” /A 48-year-old woman.

### Suggested solutions

The relatives wanted the government to be more active regarding the problems with alcohol. Stricter laws, and restrictions to reduce the availability of alcohol were wanted as well as penalties for those not following the rules. The government should sensitize people and they should build more rehab centres and put one in each district of Uganda. There was a wish to integrate hospitals with traditional healers. Community leaders should be aware of, and talk about, the dangers of alcohol, and local chairpersons should have one-to-one meetings with alcoholics. The relatives of the persons with AUD wished that they would talk about their problems to family, doctors, previous alcoholics, and friends. Schools, workplaces, and the government should be involved. They should create jobs for persons with AUD in the community.

“The Government could stop the selling of those liquids on the street, then it would be inaccessible, and some could not afford it.”

“People in the community need to be sensitized, how to help instead of marginalizing them.” / A 35-year-old man.

## Discussion

### Major findings

In this qualitative interview study, we explored EMs for AUD among patients and relatives at a clinical setting in Uganda. The most dominant themes were cultural, political, environmental, spiritual and biological; perceived to cause AUD by both patients and relatives. Children were considered to be affected in a particularly negative way by the alcohol consumption.

The findings of our study correspond with other studies from Butabika hospital regarding causes of drinking alcohol. Significant stress, secondary to early parental loss and job difficulties was observed [4]. Alcohol dependence could be a reaction to chronic illnesses such as HIV. Pooling resources and drinking in a group made alcohol more affordable, encouraging people to drink as much as they wanted. Drinking was influenced by the parents’ use of alcohol in the home when the study participants were growing up. Some parents brewed alcohol to generate income. Often, they would offer alcohol to their children, or the children would steal the alcohol [4]. Another study from Uganda showed how some parents administered alcohol to children as medicine, with a belief that it cures cough, flu, malaria and kills intestinal germs [25]. Parents could proudly introduce their children to alcohol, especially the boys, and were happy to have their sons drink, just like them. A recent study reported alcohol abuse or dependence among 5–8-year-old children living with their parents in Uganda [26]. Daily drinking and easy access through close family members was reported. Access to homemade brew was the most common source. Social vulnerability and poverty were seen as driving factors for child alcoholism. Many of the homes had stories of divorce, domestic violence, financial constraints, and violence. Some of the children complained of anxiety and trauma, such as witnessing violence and death, and some of the children used alcohol as ‘self-medication’ in their difficult circumstances, saying *“I feel good when I drink”* [26]. Another study showed that there is a significant association between alcohol initiation before 13 years of age and problem drinking among youth in Uganda [27]. These findings underscore the need for interventions and strict alcohol controls as an important policy strategy for reducing alcohol use and its dire consequences among vulnerable youth. Half of the admissions to the Ugandan National Mental Referral Hospital are young people with alcohol and substance use disorders [28]. A significantly higher alcohol use among men than women has been reported in Uganda and elsewhere [29, 30]. For example, at Butabika Mental Hospital, males were the majority of the patients treated for AUD [31].

### Culture and spirituality

There is not much research about EMs and AUD in LMICs. Most studies are from HIC. We are elucidating the conditions in one Ugandan clinical setting, and we could not find any comparable studies from SSA. It is evident that alcohol is part of the Ugandan culture and our results mirror what is seen in the literature. Alcohol was reported to be an integral part of ceremonies, such as naming children, marriage, funerals, judicial processes, and legal contracts. Hard liquor was introduced in the slave trade, and subsequent urbanization led to the development of public drinking establishments, introducing routine social drinking as part of modern culture in many African settings [32]. Participants in our study explained that there is a common belief in their culture that a spirit is drinking through the person. On the other hand, most participants in our study believed that religion could help in abstaining from alcohol. According to another study, EMs of mental disorders in lower income countries place less blame on the individual and the family by attributing causes of mental illnesses to external factors beyond the individual's control, such as God's will, Karma, or other supernatural entities [33]. Participation in religious rituals, attending religious services, praying, and discussion of religious beliefs with others have resulted in lowering the odds of drinking at unhealthy levels. As unhealthy drinking is common in Uganda, churches and religious institutions that facilitate religious behaviour may be able to play a useful role in promoting and maintaining reductions in alcohol use [6]. There is abundant evidence of the impact of religiosity and spirituality (R/S) on physical and mental health [34]. Studies have noted that R/S is associated with a better quality of life, lower rates of substance abuse, anxiety, suicide, and depression, as well as various other health benefits.

Some patients in our study talked about frequent visits to AA meetings and they had generally positive experiences. There is not much research regarding spirituality and EMs for AUD, despite its place as the central mechanism of recovery in the AA literature [35]. Previous research reveals that despite minimal spiritual beliefs, AA may facilitate increases in spirituality/religiousness, which can aid recovery from alcohol addiction. Participation in groups like AA is likely to produce changes in spirituality, coping, abstinence self-efficacy, motivation, negative affect, social networks and change at the neurobiological level [35]. Increasing numbers of studies have used experimental designs to examine the effects of spiritual practices on alcohol use and AUD recovery, demonstrating that engaging in prayer may help reduce hazardous alcohol use [36].

### Alcohol and stigma

Social problems and stigmatization affected the patients in our study. This is likely to be recognized worldwide among alcohol addicts. The severity of public stigma varies depending on the diagnostic group [33]. Substance use disorders and schizophrenia have more stigma. Negative societal responses to people with mental illnesses may be the single greatest barrier to the development of mental health programs worldwide. In LMICs there is a broader range of EMs, including religious-magical views of causation. Rather than reducing stigmatized views, neurobiological explanations have had little or no effect on social intolerance and, in some cases, have deepened it showing that good intentions are not sufficient to bring about desired change [33]. Using biological or professional explanations of mental illnesses, as a way of improving knowledge in LMICs, where literacy is generally poor, may be ill advised as an anti-stigma strategy. Stigma in both high- and low-income countries seems to be fuelled by misunderstandings of mental illness aetiology, stereotypic beliefs, and lack of political will to fund integrated mental health systems [33].

### Solutions

The relatives in our study suggested many solutions for the problems with alcohol consumption in Uganda and these opinions had clear support in the literature. Alcohol consumption and the alcohol-attributable burden of disease in Africa are expected to rise soon [37]. Increasing alcohol-related harm receives little attention from policymakers and from the population in general. To have a positive impact on the health of African populations, action addressing specific features of alcohol policy in the continent is needed, namely focusing on specifics linked to alcohol availability, like unrecorded and illicit production, outlet licensing, the expansion of formal production, marketing initiatives and taxation policies [37]. Attempts at regulation through government policy have not been very effective because of the influence of the alcohol industry, as well as the widespread practice of home-brewing/distilling [38].

### Clinical implications

To improve care for patients with AUD we have tried to understand why they drink and what they suggest as solutions to stop drinking. Research from LMICs will help high-income countries provide more culturally appropriate programs in their increasingly multi-cultural settings [33]. Decreasing mental illness-related stigma and the hidden burden of mental illness worldwide requires a concerted global effort. The importance of addressing R/S in clinical practice has been increasingly acknowledged by medical and educational organizations [34]. The American Psychiatric Association (APA), World Psychiatric Association (WPA), Royal College of Psychiatrists (RCP) and European Psychiatric Association (EPA) have verified the need to consider the spiritual dimension in psychiatry education, research, and clinical practice [34]. Despite the fact most physicians, including psychiatrists, agree on the importance of, and need to, assimilate R/S into clinical work, investigation of the religious/spiritual aspects of patients is rare [39, 40].

### Strengths and limitations

One strength is that the first author (HR) had the main responsibility for all parts of the study, participated in all the interviews and that the hospital staff was not present during the interviews. All interviews were conducted in English and no translator was needed. There were no dropouts during the study and the participants were willing to answer the questions. That responders received compensation might have led to those who were indecisive participating in this study. Further, that the participants might have said things they thought the interviewer wanted to hear. It is also possible that participants avoided dropping out to ensure they would receive the compensation. A limitation was that the participants were not representative for the Ugandan population since they were generally very well read and had a higher educational level than the population in general. The patients were all young men, since there were no women hospitalized at the ADU at Butabika hospital during the time for these interviews. All participants had a connection with Butabika hospital, and are thus not representative for all Uganda, even though the catchment area is the whole of the country. The relatives read vignettes that explained what type of patient the interview would be about. The core DSM-5 diagnostic criteria for AUD were included in the vignettes. The latter might not have fully corresponded to the signs that therapists and relatives noted, above all, in AUD.

## Conclusion

Explanatory models of AUD among hospitalized patients at the Butabika hospital in Uganda included biological, social, and spiritual explanations and alcohol was seen as an important part of the Ugandan culture. The relatives shared the same understanding of AUD and were concerned about the difficulties to find treatment. Patients and relatives considered children to be affected in alarming ways and change necessary. The results indicate that it is important in clinical contexts to investigate the EMs of the patients and their relatives. Knowledge about patients and relatives EM is a prerequisite for individually tailor treatment interventions. A better understanding of the patients´ EMs can improve the anamnesis and thus increase confidence in the clinician. In the long run, this can lead to better compliance with treatment and ameliorated outcomes. Since EMs might differ between patient and clinician it can be helpful to explore the patients´ thoughts and customize treatment and planning explanations. To help addicted individuals and their relatives in an optimal manner more research is required within different cultural contexts to better our understanding of AUD in a global perspective.

There is a notable lack of knowledge about EMs regarding AUD in Africa. This study was important to elucidate differences and similarities between how AUD is understood and related to.

## Data Availability

The datasets used and/or analysed during the current study are available from the corresponding author on reasonable request.

## Abbreviations

AA: Alcoholics anonymous
ADU: Alcohol and drug unit
AUD: Alcohol use disorder
EMIC: Explanatory model interview catalogue
EM: Explanatory model
HIV: Human immunodeficiency virus
LMICs: Low-middle-income countries
NGO: Non-governmental organization
PHC: Primary health care
SD: Standard deviation
SSA: Sub-Saharan Africa
USD: United States dollar
WHO: World Health Organization
